# Histoplasmosis in patients living with HIV in Europe: review of literature

**DOI:** 10.3389/fmicb.2024.1418530

**Published:** 2024-06-27

**Authors:** Dimitra Kontogiannis, Andrea Di Lorenzo, Drieda Zaçe, Domenico Benvenuto, Martina Moccione, Gianmarco Muratore, Maria L. Giacalone, Giulia Montagnari, Laura Carnevale, Tiziana Mulas, Luigi Coppola, Laura Campogiani, Loredana Sarmati, Marco Iannetta

**Affiliations:** ^1^Department of Systems Medicine, Infectious Disease Clinic, Tor Vergata University, Rome, Italy; ^2^Infectious Disease Clinic, Policlinico Tor Vergata, Rome, Italy

**Keywords:** histoplasma, acquired immunodeficiency syndrome, endemic, progressive disseminated histoplasmosis, dimorphic fungal pathogens

## Abstract

*Histoplasma capsulatum (*var. *capsulatum Hcc and duboisii Hcd)*, is a dimorphic fungus that causes histoplasmosis. It usually affects people coming from endemic areas, causing a variety of clinical manifestations up to progressive disseminated histoplasmosis (PDH), especially among people living with HIV (PLWH). We conducted a systematic review to assess histoplasmosis burden of PLWH in Europe. The review follows PRISMA guidelines, with protocol registered in PROSPERO (CRD42023429779). Seventy-eight articles were selected, including 109 patients (32 women). On overall, median age was 37 years. Forty-six patients were Americans, 39 Africans, 17 Europeans, 5 Asians, in 2 cases nationality was not specified. Cases were mainly diagnosed in Italy (28.4%), France (17.3%) and Spain (17.4%), with a north–south gradient. Six cases lacked epidemiologic links with endemic areas. Concerning CDC HIV staging at diagnosis, the information was available for 60 PLWH (55%) and all subjects were at stage C3 except for two subjects at stage B3. PDH was the AIDS-presenting illness in 39 patients. Most patients had a PDH (80.7%); other common extrapulmonary forms were isolated cutaneous histoplasmosis (7.3%), or lymphatic localization (2.7%). In 30 cases, the diagnosis was made by analyzing only one sample. For the remaining 79 cases, multiple samples were collected from each patient. Regarding the biological sample more frequently used for the diagnosis of histoplasmosis, bronchoalveolar lavage sample was taken from 39 patients, and tested positive in 51.3% of cases; 36 patients underwent a skin biopsy which was positive in 86.1% of cases and 28 patients performed bone-marrow biopsy, which led to the diagnosis of histoplasmosis in 92.9% of cases. The identification of *Histoplasma capsulatum* was available in 97 PLWH through examination of different samples: *Hcc* and *Hcd* were identified in 89 and 8 PLWH, respectively. Concerning therapies, 67.9% were treated with liposomal amphotericin B, 18.3% with itraconazole, 10 died pre-treatment. The overall mortality rate was 23.6%. Non-survivors exhibited more frequently gastrointestinal symptoms (*p* = 0.017), while cutaneous signs correlated with better survival (*p* = 0.05). Untreated patients faced higher mortality (*p* < 0.001). Histoplasmosis should be considered amongst opportunistic infection in PLWH, even in Europe, especially if patients originate from or have travelled to endemic areas.

**Systematic review registration:** The registration number is CRD42023429779.

## Introduction

Histoplasmosis is a mycotic infection caused by a dimorphic fungus belonging to the *Histoplasma complex*, which encompasses at least eight clades according to T. Kasuga phylogenetic analysis (Kasuga et al., 2003). Traditionally, *Histoplasma capsulatum* was classified in three varieties, var. capsulatum *Hcc* and var. duboisii *Hcd*, which are human pathogens and var. farciminosum, which is a horse pathogen. Although less accurate compared to the phylogenetic classification, in this review we kept the classification of *Histoplasma capsulatum* in var. capsulatum and var. duboisii, considering that most of the papers did not report the genetic classification of *Histoplasma* isolates. *Hcc* is the most common variant worldwide, and *Hcd* is mostly reported in Central and Western Africa (Knox and Hage, 2010; Teixeira et al., 2016). *Hcc* causes the classical or small form, in which the inhaled airborne microconidia are responsible of the infection, after conversion into the yeast phase at body temperature. *Hcd* is the causative agent of the African or large form of histoplasmosis (in tissues the yeast form reaches 12–20 μm in diameter, *Vs* 2–4 μm of *Hcc*). The two variants differ in their antigenic compositions (Georgiev, 2003).

Histoplasma infection in the immunocompetent host can be asymptomatic or can cause self-limiting flu-like symptoms or pneumonia. Severe pneumonia or disseminated disease can occur during immunosuppressive conditions, such as immunosuppressive treatments or the human immunodeficiency virus (HIV) infection, in which progressive disseminated histoplasmosis (PDH) has been included among the illnesses defining the acquired immunodeficiency syndrome (AIDS) since 1987.

Traditionally, histoplasmosis is considered to be endemic primarily in the Ohio and Mississippi River valleys within the United States, in large parts of South and Central America and in Africa (Edwards et al., 1969). HIV pandemic was associated with a rise of cases of histoplasmosis worldwide, including an increasing number of cases reported from nonendemic areas (Joseph, 2006; Bahr et al., 2015). In a recent report, the World Health Organization (WHO) included *Histoplasma* in the priority fungal pathogen list (WHO, 2022). Although for years Europe was considered a non-endemic area, *Histoplasma* has been isolated in Italy from the soil of the Po River, a region where the rate of positive histoplasmin skin test is 1.2% in the local population. In a recent revision of *Histoplasma* epidemiological maps, Italy is listed among the likely hyperendemic areas (Ashraf et al., 2020).

As for diagnosis, direct microscopic examination is a rapid technique, but culture of tissue samples or body fluids remains the gold standard. However, *Histoplasma* can take up to 6 weeks to grow in culture, which may delay diagnosis if used as the sole method. Cytology or histopathology can provide quicker results although with suboptimal sensitivity. Histological examination typically reveals pathological features such as caseous or non-caseous granulomas and narrow-based yeast forms within tissues or engulfed by macrophages. Antigen detection is a rapid and non-invasive method with high sensitivity, although its application is limited by the restricted availability and local distribution of commercial diagnostic kits. Polymerase chain reaction (PCR) methods offer a swift diagnosis, applicable to various tissue and fluid samples, and effectively distinguish histoplasmosis from other fungal infections (Hage et al., 2015).

According to the latest WHO guidelines on “Diagnosing And Managing Disseminated Histoplasmosis Among People Living With HIV,” the recommended treatment strategies include administration of liposomal amphotericin B or itraconazole. With both regimens, treatment starts with an induction phase followed by a long maintenance phase, necessary to effectively suppress residual infection and prevent relapse. The ideal duration of maintenance therapy has not been yet established (© Pan American Health Organization and World Health Organization, 2020). The latest European AIDS Clinical Society (EACS) guidelines, published in 2023, recommend to continue the maintenance phase for at least 12 months and until CD4 count reaches value over 150 cells/μL, HIV viremia is permanently undetectable for at least 6 months with negative fungal blood cultures (EACS, 2023).

**Figure 1:**
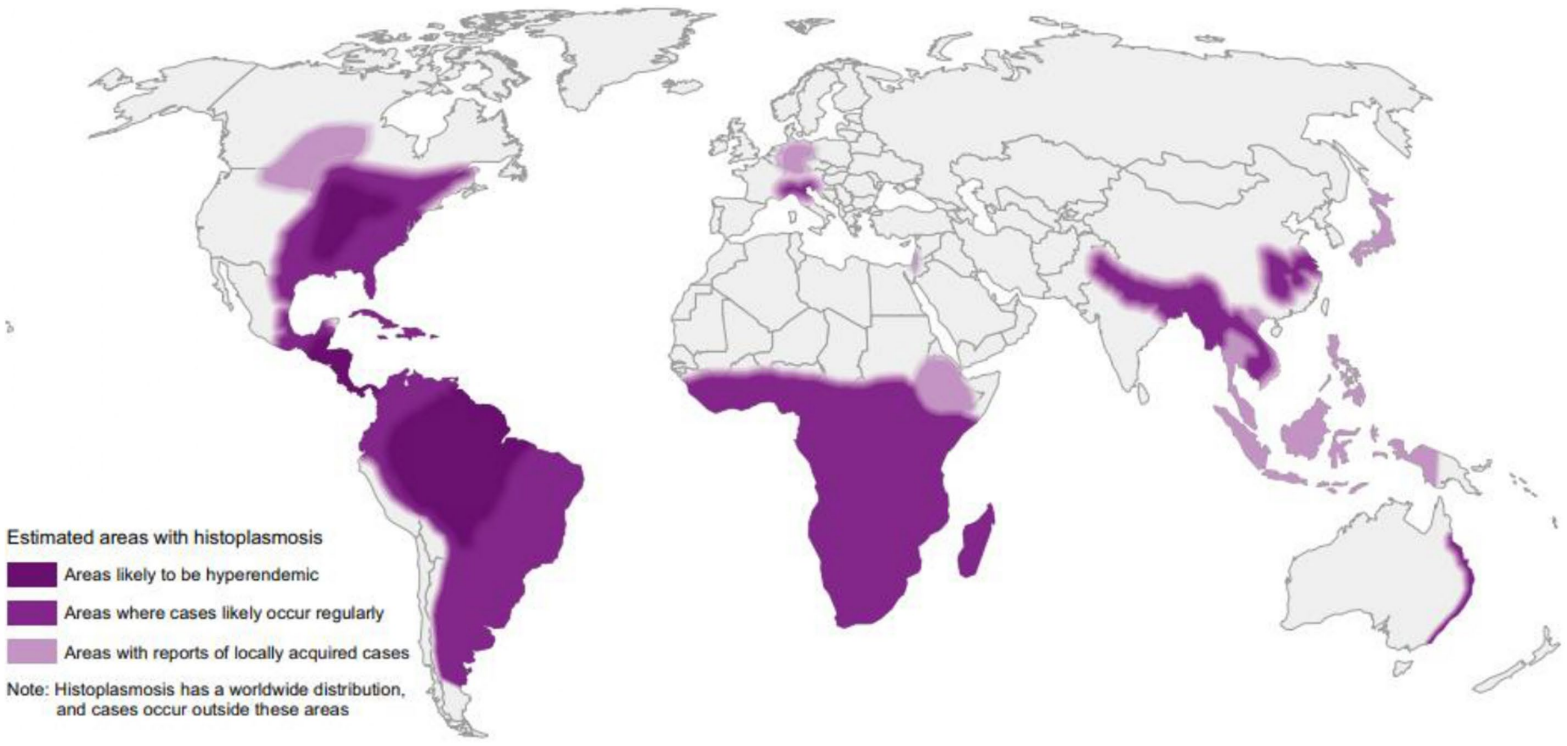
World map estimating regions most likely to have histoplasmosis based on the recent review “Re-drawing the Maps for Endemic Mycoses” by Ashraf et al. (2020). Published with permission. Licensed under CC BY 4.0 (https://creativecommons.org/licenses/by/4.0).

As previously stated, histoplasmosis was considered endemic in the Ohio and Mississippi River valleys of the United States, in South and Central America and in Africa (Edwards et al., 1969; Bahr et al., 2015). However, cases of autochthonous histoplasmosis in areas previously thought to be “non-endemic” are increasing (Schwartz et al., 2019), demonstrating a wider geographical distribution of *Histoplasma* endemic areas (Figure 1; Joseph, 2006; Ashraf et al., 2020).

The aim of this work was to carry out a systematic review of the literature, to evaluate the actual burden of histoplasmosis in PLWH in Europe.

## Methods

The systematic review of the literature was conducted and reported according to the Preferred Reporting Items for Systematic Reviews and Meta-Analyses statement (Page et al., 2021). The protocol was published in PROSPERO with registration number CRD42023429779.

### Search strategy

The electronic databases of Web of Science, PubMed and Scopus were searched to retrieve potential eligible articles, published until June 13th, 2023. A search string for PubMed was structured consisting of Medical Subject Headings terms and keywords such as HIV, Histoplasma Infection and Europe. The search in PubMed was restricted to only humans. No other restrictions were used. The search string was adapted for use in the other two electronic databases. The full search strategy for all databases can be found in online Supplemental material S1.

### Inclusion/exclusion criteria

Articles that reported data on patients with HIV and histoplasmosis infection diagnosed in Europe were included. Systematic reviews, non-empirical studies, conference abstracts, editorials, commentaries, book reviews and abstracts not accompanied by a full text were excluded. Furthermore, animal and modelling studies were excluded.

For the purposes of this review, any infection caused by *Histoplasma capsulatum* affecting any anatomical site and diagnosed through a culture test, PCR, rapid test or histology was considered a histoplasmosis case. PDH was considered when *Histoplasma capsulatum* was identified in blood cultures or bone marrow or the fungus was identified in at least two different body sites by any diagnostic method.

### Study selection

All studies retrieved from the search strategy were imported to RAYYAN QCRI software, duplicates were removed (Ouzzani et al., 2016). Four researchers (DK, MM, GM and DZ), divided in two groups, independently performed the first screening based on titles and abstracts. Discrepancies were resolved by a fifth researcher (ADL). In a second step, studies with full texts available were entirely read by four researchers (GM, MLG, DB, and ADL) to decide the final articles to include in the review. When it was not possible to retrieve any full text online, the corresponding authors of the articles were contacted. Due to its relevance on the topic, the bibliography of the systematic review published in 2021 by Antinori et al. (2021). was analyzed, and additional papers were included.

### Data extraction and analysis

Data extraction was performed by four researchers (GM, DK, DB, and ADL). A dedicated data extraction form was used to retrieve the following information for each eligible study:

1. study identification and characteristics (first author, title, publication year, study design);
2. histoplasmosis cases’ characteristics (age, gender, country of origin, country of diagnosis, travels to endemic zones, infection localization, clinical symptoms);
3. diagnostic method and type of sample;
4. HIV co-infection data (time from HIV diagnosis to histoplasmosis diagnosis, Centers for Disease Control and Prevention (CDC) class at HIV diagnosis, risk factor for HIV infection, CD4 count, CD4/CD8 ratio and HIV viremia at histoplasmosis diagnosis);
5. histoplasmosis treatment and outcome.

A descriptive analysis of patients’ data deriving from the included studies was done. Patients were then grouped in survivors and non-survivors. All analyses were performed using the software JASP (version 0.17.0 JASP Team, 2019).

### Quality assessment

Two researchers (DK and ADL) independently conducted the methodological quality assessment, based on the study designs. Disagreements were resolved by discussion with a third researcher (DZ). We used ‘Study Quality Assessment Tools’ of the National Heart, Lung and Blood Institute for case series/case report studies (National Heart, Lung, and Blood Institute, 2013).

## Results

**Figure 2:**
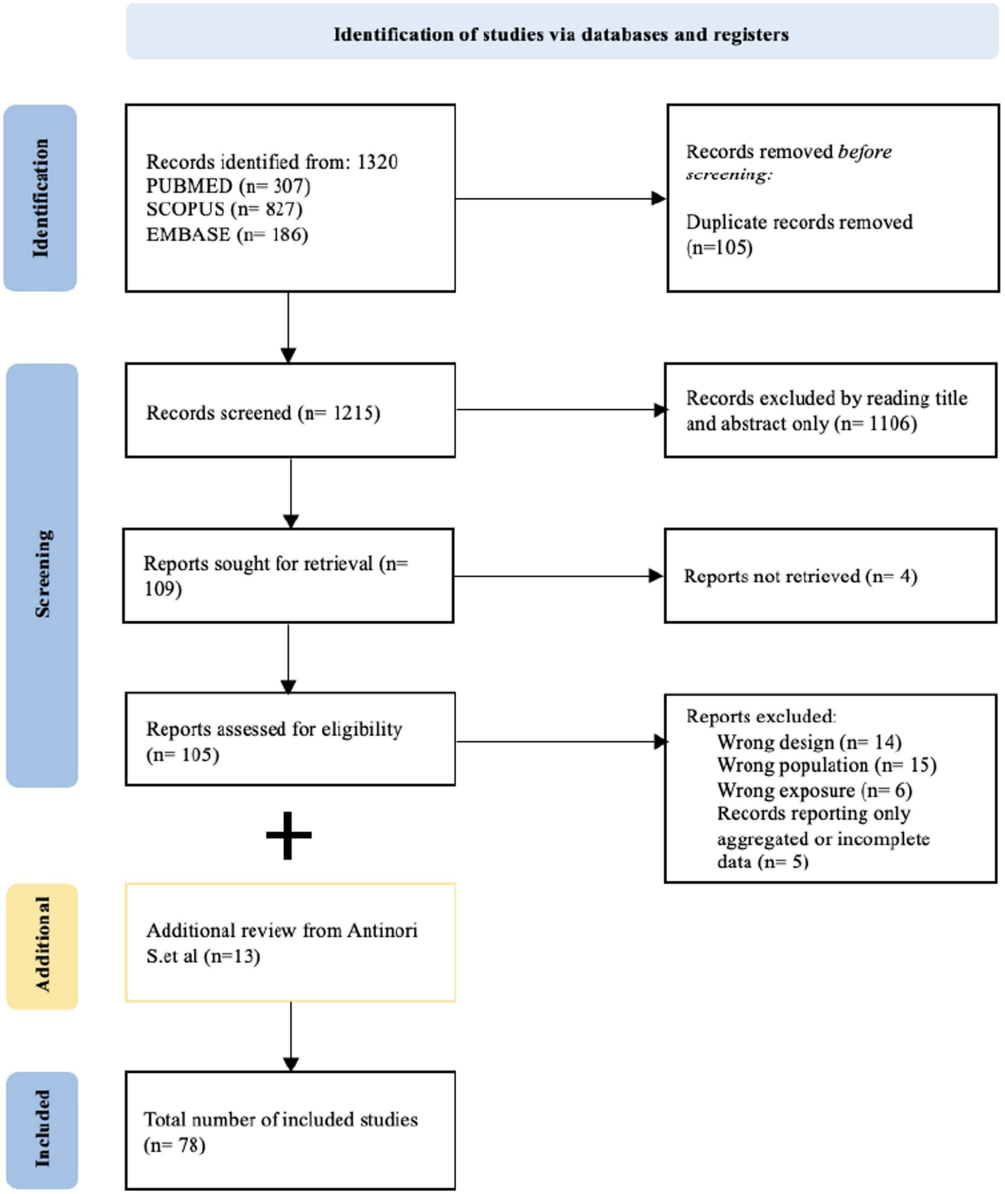
PRISMA flowchart of article selection process.

A total of 1,320 articles was identified (307 from PUBMED, 827 from SCOPUS and 186 from EMBASE). After deduplication, 1,215 articles were included; 1,106 articles were excluded by reading the title and the abstract only, not meeting the pre-defined inclusion criteria. At the end of the review process, 65 articles were included (52 case reports and 13 case series, with additional 37 cases) (Dietrich et al., 1987; Smith et al., 1989; Rockstroh et al., 1991; Boulard et al., 1994; Manfredi et al., 1994; Wockel et al., 1994; Hofman et al., 1995; Trylesinski et al., 1995; Eichmann and Schär, 1996; Grosse et al., 1997; Angius et al., 1998; Schöfer and Baur, 1998; Guex, 1999; Knapp et al., 1999; Rieg et al., 1999; Antinori et al., 2000, 2006; Farina et al., 2000, 2005; Bilkenroth and Holzhausen, 2001; Rivasi et al., 2001; Carey et al., 2002; Molina et al., 2002; Rickerts et al., 2002; Calza et al., 2003; Lo Cascio et al., 2003; Crommentuyn et al., 2004; Faggian et al., 2004; Terrancle de Juan et al., 2004; Ferry et al., 2005; Matulionyte et al., 2005; Buhk et al., 2006; Gil-Brusola et al., 2007; Loulergue et al., 2007; Albrecht et al., 2008; Débat Zoguéreh et al., 2008; Peppa et al., 2008; De Lavaissière et al., 2009; Norman et al., 2009; Pellaton et al., 2009; Ala-Kauhaluoma et al., 2010; Pineau et al., 2010; Borges-Costa et al., 2011; Bourgeois et al., 2011; Inojosa et al., 2011; Navascués et al., 2011; Vaid and Patel, 2011; Carena Smuckler et al., 2012; Escher et al., 2012; Scarlata et al., 2012; Sharma et al., 2012; Navarro et al., 2013; Shah et al., 2013; Stevenson and Taylor, 2014; Therby et al., 2014; Delfino et al., 2015; Stete et al., 2015; Lehur et al., 2017; Sánchez et al., 2017; Chroboczek et al., 2018; Evrard et al., 2018; Zanotti et al., 2018; Lebowitz et al., 2019; Ghorra et al., 2022). The bibliography of a recent systematic review on histoplasmosis, published in 2021 by Antinori et al. (2021). was screened, and additional 13 articles with 20 cases described (9 case reports and 11cases from 4 case series) were included (de José Gómez et al., 2005; Pistone et al., 2005; Breton et al., 2006; Peters et al., 2006; Del Mar et al., 2007; Débat Zoguéreh et al., 2008; Gomez-Moyano et al., 2011; Sanmani et al., 2011; Amadori et al., 2015; Gómez-Espejo et al., 2017; Bosch-Nicolau et al., 2019; Engelmann et al., 2019; Papalini et al., 2019), reaching a final population of 78 articles, describing 109 patients (Figure 2).

**Figure 3:**
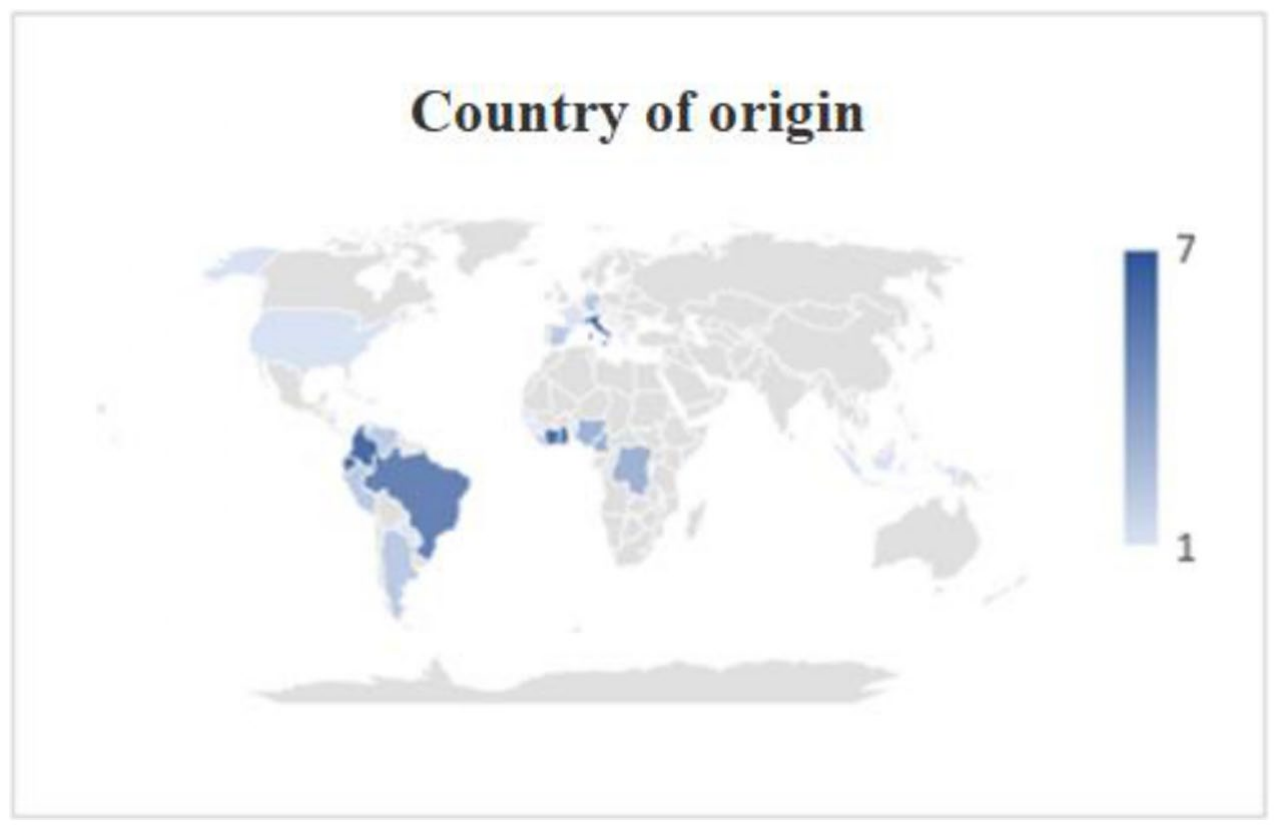
Origin of patients. It is noted that, in addition to patients from areas considered classically endemic, cases of Italian (7), Spanish (2) and German (2) nationality were reported.

**Figure 4:**
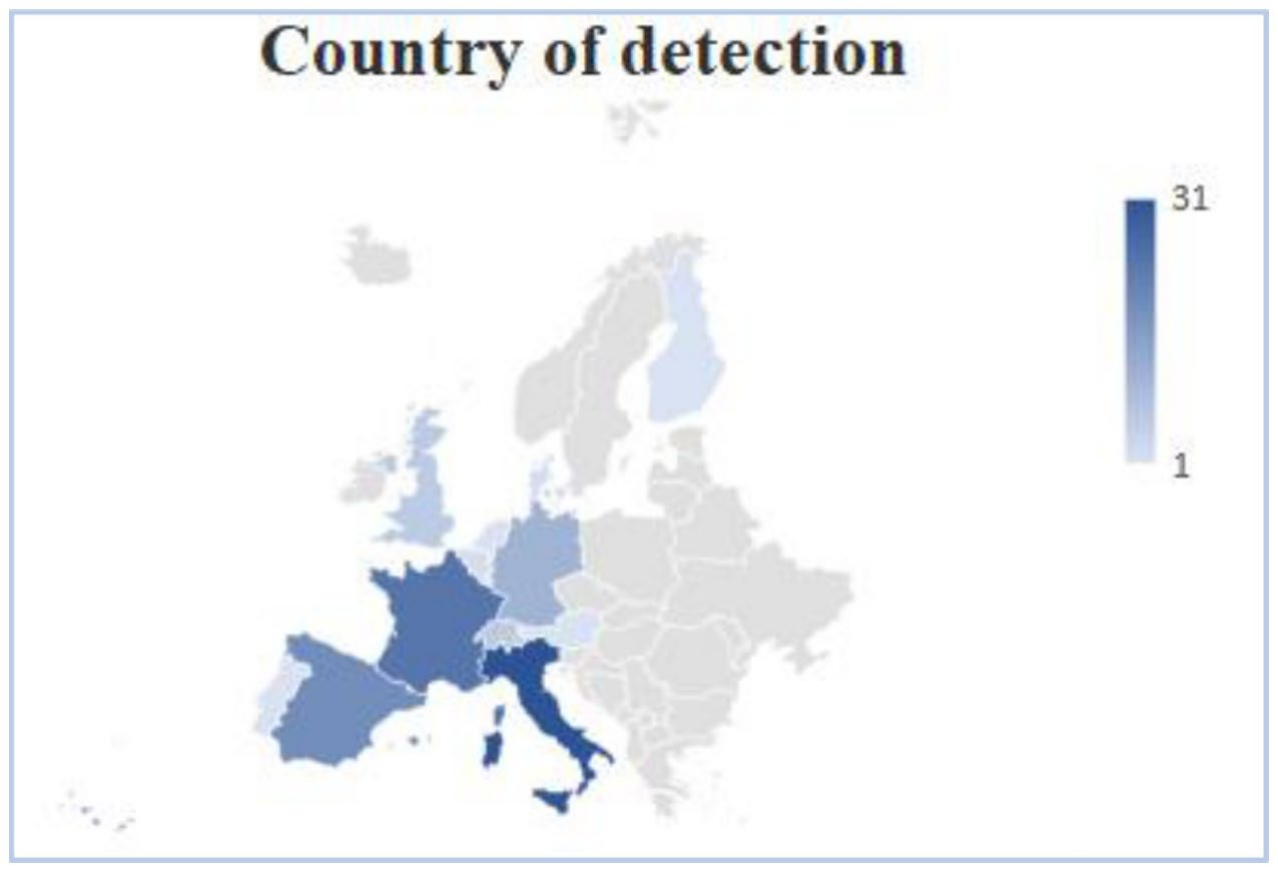
Country of identification of histoplasmosis in Europe, histoplasmosis cases were mainly identified in Italy, France and Spain, with an apparent north–south gradient. Other countries were Germany, Switzerland, United Kingdom, Belgium, Denmark, Netherlands, Austria, Finland, Portugal and in five cases the data was not available.

Seventy-one out of 109 patients were males (65.14%), 32 were females (29.59%), and 6 were transgender women (5.5%); overall the median age was 37 years [interquartile range (IQR) 32–43] (Table 1). Forty-six patients were Americans (42.2%), 39 Africans (35.78%), 17 Europeans (15.6%), 5 Asians (4.59%), in two cases the nationality of origin was not specified (Table 1 and Figure 3). Histoplasmosis cases were mainly diagnosed in Italy (31 patients, 28.4%), France (21 patients, 17.26%) and Spain (19 patients, 17.43%), with an apparent north–south gradient (Table 1 and Figure 4). Among the patients from non-endemic areas, 6 patients did not report an epidemiologic link with hyperendemic areas: two Italian and one Spanish patient explicitly reported no travel to endemic areas, while for two Italians and one patient from Serbia, there was insufficient information regarding recent travel history.

**Table 1:** Overall population characteristics.

Overall population – 109 patients	
Age – median years [IQR]	37 [32–43]
Sex: *n*, (%)	
Males	71 (65.14)
Females	32 (29.59)
Transgender	6 (5.5)
Continent of origin: *n*, (%)	
America	46 (42.2)
Africa	39 (35.78)
Europe	17 (15.6)
Asia	5 (4.59)
NA	2 (1.83)
CDC state at HIV diagnosis (data for 60 pts): *n*, (%)	
C3	58 (96.7)
B3	2 (3.3)
Time between HIV diagnosis and Histoplasma infection - median months [IQR] (data for 85 pts)	3 [0–48]
Country of histoplasmosis diagnosis: *n*, (%)	
Italy	31 (28.4)
France	23 (21.3)
Spain	19 (17.4)
Germany	11 (10.1)
Switzerland	6 (5.5)
United Kingdom	6 (5.5)
Belgium	2 (1.8)
Denmark	2 (1.8)
Netherlands	1 (0.9)
Austria	1 (0.9)
Finland	1 (0.9)
Portugal	1 (0.9)
NA	5 (4.6)
Median HIV-RNA [IQR] at Histoplasmosis diagnosis (data for 55 pts)	199,526 copies/ml [38690–750,000]
Median CD4 cell count at Histoplasmosis diagnosis [IQR] (data for 106 pts)	19 [8–40]
Histoplasma species identified (data for 97 pts): n, (%)	
Hcc *(capsulatum var. capsulatum)*	89 (88.1%)
Hcd *(capsulatum var. duboisii)*	8 (7.9%)
NA	12 (11.9%)
Histoplasmosis form: n, (%)	
Disseminated	88 (80.7)
Cutaneous	8 (7.3)
Lymphatic	3 (2.8)
Gastrointestinal	2 (1.8)
Pulmonary	2 (1.8)
Other	6 (5.5)
Symptoms: *n*, (%)	
Fever	91 (83.5)
lymphopenia, anemia, or thrombocytopenia	71 (65.1)
Weight loss	55 (50.5)
Lymphadenopathy	51 (46.8)
Hepatosplenomegaly	47 (43.1)
Gastrointestinal symptoms	46 (42.2)
Skin lesions	45 (41.3)
Respiratory symptoms	43 (39.4)
Neurological symptoms	15 (13.8)
Treatment: n, (%)	
Liposomal amphotericin	74 (67.9)
Itraconazole	20 (18.3)
Fluconazole	2 (1.8)
NA	13 (11.9)
Outcome: *n*, (%)	
Discharged	81 (74.3)
Died	25 (22.9)
NA	3 (2.7)

HIV, human immunodeficiency virus; CDC, Centers for Disease Control and Prevention; NA, not available.

CDC staging at HIV diagnosis was reported for 60 (55%) patients: all were at stage C3 of the CDC classification except two patients that were at stage B3 (Schöfer and Baur, 1998; Knapp et al., 1999). When reported (84 patients), the median time elapsed between HIV diagnosis and *Histoplasma* infection was 3 months [IQR 0–48]. PDH was the AIDS-presenting illness in 39 patients. Regarding the immunovirological status at the diagnosis of histoplasmosis, the median HIV-1 viremia, available for 55 patients, was 199,526 copies/ml [IQR 38690–750,000]; CD4 T-cell absolute count was available for 106 PLWH and was <200 cells/uL in all cases. CD4/CD8 ratio, available for 16 patients, was 0.085 [IQR 0.02–0.185]. Two patients had a HIV-2 infection. Risk factors for HIV infection were reported only in 30 cases (27.5%) and in 70% (21 patients) was represented by sexual intercourses.

The majority of patients had a PDH (88 patients, 80.7%), other common extrapulmonary forms were isolated cutaneous histoplasmosis (8 patients, 7.3%), or lymphatic localization (3 patients, 2.7%). All patients were symptomatic, most of them had fever, lymphopenia, anemia, thrombocytopenia and weight loss (Table 1). Forty-two out of 109 patients with histoplasmosis had a concomitant opportunistic infection (38.5%): the most common were invasive candidiasis (10 patients, 23.8%), *Pneumocystis jirovecii* pneumonia (9 patients, 21.4%), and mycobacterial infection (8 patients, 19% - 4 atypical, 4 by *Mycobacterium tuberculosis*) (Table 2).

**Table 2:** Concomitant opportunistic infections.

Concomitant opportunistic infections	Number of patients (42/109)
Invasive cadidiasis (%)	10 (23.8)
Pneumocystis jirovecii pneumonia (%)	9 (21.4)
Cytomegalovirus disease (%)	7 (16.6)
MAC (%)	4 (9.5)
Tuberculosis (%)	4 (9.5)
Kaposi sarcoma (%)	4 (9.5)
Herpes simplex 2 (%)	3 (7.1)
Toxoplasmosis (%)	2 (4.8)
Strongyloidiasis (%)	2 (4.8)
HCV (%)	2 (4.8)
Molluscum contagiosus (%)	1 (2.4)
HTLV1 (%)	1 (2.4)
Cystoisosporiasis (%)	1 (2.4)
HBV (%)	1 (2.4)
Fusarium verticillioides (%)	1 (2.4)
Syphilis (%)	1 (2.4)
Cryptococcosis (%)	1 (2.4)
Giardiasis (%)	1 (2.4)
Leishmaniosis (%)	1 (2.4)
Aspergillosis (%)	1(2.4)
Coccidioidomycosis (%)	1 (2.4)
Chagas disease (%)	1 (2.4)

MAC, *Mycobacterium avium* complex; HSV, herpes simplex virus; HCV, hepatitis C virus; HTLV1, Human T-lymphotropic virus 1; HBV, Hepatitis B virus.

In our review, we analyzed a total of 256 samples from 109 patients. In 30 cases (27.5%), the diagnosis was made by analyzing only one sample. For the remaining 79 cases (72.5%), multiple samples were collected from the same patient, with a median of 2 samples per patient [IQR 2–3], ranging from a minimum of 2 to a maximum of 8 samples. Among these 79 patients, 23 cases had a single positive test that led to the diagnosis. When the same sample was analyzed using different methods, we considered culture as the gold standard for diagnosis. In total, 93 diagnoses were made using culture methods (85.3%), 9 using PCR (8.6%), 5 using histological methods (4.6%), 1 using a rapid test (0.9%), and in one case the method was not specified.

Regarding the samples more frequently collected, a bronchoalveolar lavage sample was taken from 39 patients (35.7%), of which 20 resulted positive for *Histoplasma capsulatum* (51.3%) identified from culture (16, 80%), microscopy (2, 10%) or PCR (2, 10%). Thirty-six patients (33%) underwent a skin biopsy which resulted positive in 31 cases (86.1%) mostly from culture (23, 74.2%), and 28 patients (25.7%) performed a bone-marrow biopsy of which 26 resulted positive (92.9%) with a positive culture in 15 cases (75%) (Table 3). In addition, 27 blood cultures (24.8%) tested positive for *Histoplasma*. The identification of *Hc* was available in 97 patients through examination of different samples. In 89 patients *Hcc* was identified (80.7%) while 8 cases had *Hcd* infection (7.3%), 12 cases (11%) did not report the isolated *Histoplasma capsulatum* species. As reported in WHO Guidelines, antibody tests are usually not helpful for diagnosing PDH among PLWH (Pan American Health Organization and World Health Organization, 2020). In our review only six papers (Rockstroh et al., 1991; Farina et al., 2000; Faggian et al., 2004; Ala-Kauhaluoma et al., 2010; Shah et al., 2013; Sánchez et al., 2017) reported *Histoplasma*-specific serological status with 3 positive subjects (50%).

**Table 3:** Diagnostic samples collected and positivity rate of the different test performed.

Diagnostic samples: *n*, (%)	Collected	Positivity	Culture	Microscopy	PCR
Bronchoalveolar lavage	39/109 (35.8)	20/39 (51.3)	16/20 (80)	2/20 (10)	2/20 (10)
Cutaneous biopsy	36/109 (33)	31/36 (86.1)	23/31 (74,2)	5/31 (16,1)	3/31 (9,7)
Bone marrow biopsy	34/109 (31.2)	20/34 (58.8)	15/20 (75)	4/20 (20)	1/20 (5)
Lymph node biopsy	28/109 (25.7)	26/28 (92.9)	19/26 (73,1)	5/26 (19,2)	2/26 (7,7)
Gastrointestinal biopsy	22/109 (20.2)	16/22 (72.7)	11/16 (68,8)	3/16 (18,8)	2/16 (12,5)
Bone marrow aspiration	20/109 (18.3)	15/20 (75)	15/15 (100)	/	/
Pulmonary biopsy	13/109 (11.9)	10/13 (76.9)	5/10 (50)	5/10 (50)	/
Cerebrospinal fluid	12/109 (11)	2/12 (16.7)	2/2 (100)	/	/

Regarding treatment, for two cases the information was not available (Grosse et al., 1997; Terrancle de Juan et al., 2004), one patient did not start the treatment because he self-discharged against medical advice (Rivasi et al., 2001) and 10 patients died before starting treatment. For the remaining cases, 74 out of 96 patients (77.1%) were treated with liposomal amphotericin B, 20/96 with itraconazole (20.8%), 2/96 with fluconazole (2.1%). Overall, 81 patients were discharged alive, 25 patients died during the hospitalization, and 3 patients were lost at follow-up, showing an overall mortality rate of 23.6% (Table 4).

**Table 4:** Comparison between survivors and non-survivors.

	Survivors 81 patients	Non-survivors 25 patients	*p*-value
Age median years [IQR]	36 [32–43]	37 [31–42]	0.94
Sex: Male/Female/Transgender	55/21/5 (67.9/25.9/6.2)	13/11/1 (52/44/4)	0.225
Hcc/Hcd	64/6 (91.4/8.6)	23/1 (95.8/4.2)	0.478
Disseminated/localized	66/15 (81.5/18.5)	21/4 (84/16)	0.774
Delta diagnosis HIV/Hc median months [IQR]	3 [0–60]	0 [0–12]	0.225
CDC stage B3/C3	1/44 (2.2/97.8)	1/13 (7.1/92.9)	0.374
HIV-RNA at Hc diagnosis median value [IQR]	234,594 [30660–750,000]	159,763 [105000–1,000,000]	0.578
CD4/CD8 Hc diagnosis median value [IQR]	0.110 [0.02–0.18]	0.045 [0.02–0.16]	1
Symptoms Yes/No			
Respiratory	33/48 (40.7/59.3)	9/16 (36/64)	0.672
Gastrointestinal	30/51 (37/63)	16/9 (64/36)	**0.017**
Fever	65/16 (80.2/19.8)	24/1 (96/4)	0.061
Neurological	11/70 (13.6/86.4)	4/21 (16/84)	0.762
Cutaneous	36/45 (44.4/55.6)	6/19 (24/76)	**0.05**
Weight loss	43/38 (53.1/46.9)	12/13 (48/52)	0.656
Hepatosplenomegaly	37/44 (45.7/54.3)	10/15 (40/60)	0.617
Lymphadenopathy	40/41 (49.4/50.6)	11/14 (44/56)	0.638
Pancytopenia	52/29 (64.2/35.8)	18/7 (72/28)	0.471
Treatment regimen LA/F/I/No therapy	61/2/17/1 (75.3/2.5/21/1.2)	13/0/2/10 (52/0/8/40)	**<0.001**

Logistic regression			
	Odds ratio	95% C.I.	***p*-value**
Gastrointestinal symptoms	0.298	−2.357 to –0.068	**0.033**
No therapy	0.018	−6.228 to –1.812	**<0.001**

Hcc, *Histoplasma capsulatum* var. *capsulatum*; Hcd, *Histoplasma capsulatum* var. *duboisii;* HIV, human immunodeficiency virus; CDC, Centers for Disease Control and Prevention; LA, liposomal amphotericin B; I, itraconazole; F, fluconazole; C.I., confidence interval. Outcome level “survived” coded as class 1. Bold values reported *p*-value<0.05.

After stratifying patients into survivors and non-survivors, gastrointestinal symptoms were more frequent in non-survivors (*p*-value: 0.017); cutaneous signs were significantly associated with greater survival (*p*-value: 0.05) (Table 4). Moreover, a notable correlation existed between receiving the treatment and the outcome, as those not treated had a higher mortality (*p*-value<0.001). After performing a logistic regression analysis considering the significative association of the univariate analysis, it was confirmed the association of higher mortality with gastrointestinal symptoms (*p*-value 0.033) and not receiving specific therapy for histoplasmosis (*p*-value<0.001).

## Discussion

This review aimed to assess the actual burden of histoplasmosis among PLWH in Europe and to identify symptom or demographic characteristics that might influence the outcome. Hundred and nine patients from 78 articles were included in the review. Most patients were male with a low median age (37 years), in accordance with existing literature findings (Ashbee et al., 2008; Mittal et al., 2019). This prevalence does not necessarily imply a greater susceptibility among younger individuals or males, but is rather associated with the mode of histoplasmosis transmission. Infection arises from the inhalation of the fungus present in soil, particularly when contaminated by bird and bat feces. Young males are often more engaged in outdoor activities that entail a heightened risk of fungus exposure, potentially explaining higher incidence associated with these characteristics (Ashbee et al., 2008; Mittal et al., 2019).

Only 8 (7.3%) of the 109 patients were infected with *Hcd* in our review, which aligns with its lower prevalence, particularly in PLWH (Antinori et al., 2006). *Hcd* typically manifests with cutaneous or bone involvement and is mostly reported in Central and Western Africa, primarily in rural settings. The underlying reasons for the low prevalence of *Hcd* remain uncertain; however, it may be attributed to misdiagnosis and the distinct geographic distribution of *Hcd*, which is more prevalent in rural areas, in contrast with HIV, which predominantly affects urban areas (Antinori et al., 2006).

Interestingly, in literature, a notable gradient in histoplasmosis identification is observed from southern to northern Europe. As in the review of Ashraf et al. (2020), in our review a preponderance of cases is reported in Italy. In contrast, in a survey conducted by Ashbee et al. (2008), the majority of cases were observed in Germany during the years 1995 to 1999, probably for the different time period considered. The review by Antinori et al. (2021). included studies published from 2000 to 2020, and found that 49.1% of cases of histoplasmosis in PLWH were reported in Spain, followed by France (19.3%) and Italy ranking as the third country of diagnosis, accounting for 12.3% of cases. This difference in the prevalence can be explained by the fact that they included also the review of Buitrago et al. (2006), that we excluded as it is not a primary source. The elevated prevalence observed in Spain and France can be attributed to immigration from former colonial territories to these countries, a trend supported by both our review and existing literature (Antinori et al., 2006, 2021). Eighty-five patients in the present review were of African and American origin, and of those born in non-endemic countries, 13 patients had a history of travel to Africa and America. Existing literature highlight Africa and America as areas with elevated suspected *Histoplasma* exposure (Antinori et al., 2006, 2021; Ashbee et al., 2008). In our review, nearly all patients had a history of travel to or migration from endemic countries, nevertheless it is interesting to note the presence of six autochthonous cases. For three cases, the description of their previous travel history was not clearly described (Farina et al., 2000, 2005; Escher et al., 2012), and for the remaining three, the history of previous travel in endemic country was explicitly denied (Antinori et al., 2000; Calza et al., 2003; Terrancle de Juan et al., 2004). Among these cases, four were of Italian origin, the remaining two were from Spain and Serbia. Considering reported veterinarian cases of histoplasmosis and the isolation of *Histoplasma* from the soil of the Po River, these findings collectively support the classification of Italy as a region with a low-level endemicity (Antinori et al., 2021).

Among these cases, Terrancle de Juan et al. (2004) reported a case of histoplasmosis in 2004 involving two drug-addicted brothers who shared syringes. The epidemiological link, in the form of a trip to an endemic region, was established only for the brother who deceased from disseminated histoplasmosis. In contrast, the described patient also developed PDH but lacked a clear travel history to an endemic area. A plausible hypothesis for the infection in this case involves fungemia during syringe sharing, akin to the transmission mechanism seen in other fungal infections (Carlucci et al., 2016; Levitt et al., 2020).

In our systematic review, histoplasmosis mainly occurred in advanced HIV patients (96.7% CDC C3), a median of 3 months after HIV diagnosis. Only two patients had a diagnosis of HIV in B3 stage: in the first case (Knapp et al., 1999) the time between HIV and histoplasmosis diagnosis is not specified, while in the second case (Schöfer and Baur, 1998) the patient was diagnosed with HIV 8 month previously and histoplasmosis was the first manifestation of AIDS. Unlike immunocompetent subjects, who rarely develop disseminated histoplasmosis, PLWH usually present with PDH, a finding that was also observed in the review by Ashbee et al. (2008). (97.7%, 42/43 cases), and in the systematic review by Antinori et al. (2021). (89.5%, 102/114 cases). Our review of the literature corroborates these findings, showing that histoplasmosis manifests in the clinical form of PDH in 80.7% (88/109) of PLWH. PDH was the AIDS-presenting illness in 39 newly diagnosed patients, a prevalence consistent with literature data (Antinori et al., 2021), but histoplasmosis can also present several months or even years after HIV diagnosis. Immunological status seems to be fundamental in histoplasmosis manifestation: in our systematic review, of the 106 patients with reported CD4 levels at the time of histoplasmosis diagnosis, none had CD4 levels above 200 cells/ml. In Hajjeh et al. (2001), 85% of patients had a CD4 lymphocyte count of <100 cells/mL, and in the work of Ashbee et al. (2008). the percentage of patients who had a CD4 lymphocyte count of <150 cells/mL is even higher (94.7%), confirming the preferential presentation of histoplasmosis in severely immunocompromised patients.

According to data from previous reviews (Antinori et al., 2006; Ashbee et al., 2008; Antinori et al., 2021) and from the present systematic review, a high portion of patients diagnosed with histoplasmosis present with other concomitant opportunistic infections (38.5% in our systematic review). Histoplasmosis is not often included in differential diagnosis when assessing PLWH, especially in non-endemic areas. Physicians usually prioritize screening for infections such as tuberculosis or pneumocystosis, particularly in patients presenting with respiratory symptoms. Only after treatment failure for these opportunistic infections, further investigations are warranted, eventually leading to histoplasmosis diagnosis.

Identifying *Histoplasma* poses a considerable challenge; in cases of chronic pulmonary presentation, it can be easily mistaken for conditions like tuberculosis, sarcoidosis, and blastomycosis. Similarly, when it manifests as a gastric presentation, it may mimic the appearance of a tumor (Doleschal et al., 2016). In addition, misidentification is possible in the microbiology laboratory with other organisms such as *Candida glabrata*, *Pneumocystis jiroveci, Toxoplasma gondii*, *Leishmania donovani*, *Cryptococcus neoformans* (Villareal et al., 2023).

As for the clinical presentation, fever was the most frequent symptom, present in 83.5% of cases, followed by weight loss and respiratory symptoms, described in the literature as flu-like syndrome (Ashbee et al., 2008; Antinori et al., 2021). Gastrointestinal symptoms resulted, from our analysis, associated with higher mortality (*p*-value 0.017). The reason for the higher mortality among patients presenting with gastrointestinal symptoms at onset could be explained by the fact that they can often be misdiagnosed as inflammatory bowel disease, malignancy, or other intestinal diseases leading to inappropriate therapies and delaying proper treatment (Kahi et al., 2005). Notably a case–control study of risk factors for Histoplasmosis in PLWH (Hajjeh et al., 2001) revealed an association between the presence of gastrointestinal injury and higher mortality rates. However, it is interesting to note that in endemic areas, where physicians are aware of the very high incidence of the disease and its frequent presentation as febrile diarrhoea, gastrointestinal presentations were milder in a large cohort of PLWH with histoplasmosis (Nacher et al., 2021).

In our systematic review, the diagnosis was mainly made through histopathology (91%, 81/89) examination, of which 71 also had a positive culture; only in 8 cases the diagnosis was made by culture examination alone. Histology assumes a pivotal role within the diagnostic algorithm of histoplasmosis (Antinori et al., 2006; Ashbee et al., 2008; Drak Alsibai et al., 2020; Antinori et al., 2021). Skin and lymph node biopsy analysis yielded a high positivity rate (86.1 and 92.2% respectively); conversely a bronchoalveolar lavage culture test had an unexpected low positivity rate (51.3%). In accordance with the literature, the occurrence of Central Nervous System (CNS) involvement, whether as a manifestation of disseminated disease or an isolated focal infection, was infrequent (Antinori et al., 2006), with only 16.7% positivity rate in cerebrospinal fluid culture. A patient described by Ala-Kauhaluoma et al. (2010) presented with PDH, involving CNS and with ocular localization during maintenance therapy with voriconazole. This finding underscores the importance of considering the possibility of ocular disease in such cases and making decisions regarding the most appropriate therapeutic approach. Despite some studies indicating therapeutic levels of voriconazole in the aqueous and vitreous (Hariprasad et al., 2004), the management of ocular histoplasmosis remains a complex and important aspect of patient care.

Early identification of histoplasmosis infection and early treatment are critical, as can be deduced from the high mortality rate we reported (23.6%). The 9.2% mortality rate observed prior to treatment initiation aligns with the findings of Antinori et al. (2006) that report a cumulative mortality rate of 15.2% during the induction therapy. This serves as a compelling rationale for intensifying the clinical emphasis on both diagnostic strategies and clinical management in the context of histoplasmosis.

## Conclusion

Given the elevated mortality rate, the diagnostic complexities and a potentially long latency period, it is essential to consider *Histoplasma capsulatum* infection when evaluating an HIV positive patient, even in regions not traditionally considered endemic. Also, according to our review, it may be necessary to perform multiple sampling in order to establish a diagnosis, enabling the prompt initiation of effective therapeutic measures.

## Data availability statement

The raw data supporting the conclusions of this article will be made available by the authors, without undue reservation upon request.

## Author contributions

DK: Conceptualization, Data curation, Formal analysis, Investigation, Methodology, Project administration, Resources, Software, Validation, Visualization, Writing – original draft, Writing – review & editing. AD: Formal analysis, Investigation, Methodology, Project administration, Resources, Software, Supervision, Validation, Visualization, Writing – original draft, Writing – review & editing, Data curation, Conceptualization. DZ: Conceptualization, Data curation, Formal analysis, Investigation, Supervision, Visualization, Writing – original draft, Writing – review & editing. DB: Formal analysis, Writing – original draft, Writing – review & editing. MM: Writing – original draft, Writing – review & editing. GMu: Writing – original draft, Writing – review & editing. MG: Writing – original draft, Writing – review & editing. GMo: Writing – original draft, Writing – review & editing. LCar: Writing – original draft, Writing – review & editing. TM: Writing – original draft, Writing – review & editing. LCo: Writing – original draft, Writing – review & editing. LCam: Supervision, Validation, Writing – original draft, Writing – review & editing. LS: Data curation, Methodology, Project administration, Supervision, Validation, Writing – original draft, Writing – review & editing. MI: Data curation, Methodology, Project administration, Supervision, Validation, Writing – original draft, Writing – review & editing.
