# Supplementary material for: Histoplasmosis in patients living with HIV in Europe: review of literature
Source: Front Microbiol. 2024 Jun 27;15:1418530. doi: 10.3389/fmicb.2024.1418530 (PMC11238259; doi:10.3389/fmicb.2024.1418530)
Supplement: Supplementary file 1 [file Table_1.DOCX]

Full search strategy for all databases (June 13^th^, 2023)

Pubmed (only humans) tot 307

("HIV"[Mesh] OR “Human Immunodeficiency Virus” OR “AIDS Virus” or “Acquired Immune Deficiency Syndrome Virus” OR “PLWHIV”) AND (“Histoplasma Infection” OR “Disseminated Histoplasmosis” OR “Pulmonary Histoplasmosis” OR “Histoplasma duboisii Infection” OR “African Histoplasmosis” OR “Histoplasma capsulatum Infection” OR histoplasm*)

Scopus (only articles) 827

(“Human Immunodeficiency Virus” OR AIDS OR “Acquired Immune Deficiency Syndrome Virus” OR “PLWHIV”) AND (“Histoplasma Infection” OR “Disseminated Histoplasmosis” OR “Pulmonary Histoplasmosis” OR “Histoplasma duboisii Infection” OR “African Histoplasmosis” OR “Histoplasma capsulatum Infection”) AND Europ*

Embase (only articles) 186

(“Human Immunodeficiency Virus” OR AIDS OR “Acquired Immune Deficiency Syndrome Virus” OR “PLWHIV”) AND (“Histoplasma Infection” OR “Disseminated Histoplasmosis” OR “Pulmonary Histoplasmosis” OR “Histoplasma duboisii Infection” OR “African Histoplasmosis” OR “Histoplasma capsulatum Infection”) AND Europ*
